# DLML-PC: an automated deep learning and metric learning approach for precise soybean pod classification and counting in intact plants

**DOI:** 10.3389/fpls.2025.1583526

**Published:** 2025-07-21

**Authors:** Yixin Guo, Jinchao Pan, Xueying Wang, Hong Deng, Mingliang Yang, Enliang Liu, Qingshan Chen, Rongsheng Zhu

**Affiliations:** ^1^ College of Engineering, Northeast Agricultural University, Harbin, China; ^2^ College of Arts and Sciences, Northeast Agricultural University, Harbin, China; ^3^ National Key Laboratory of Smart Farm Technologies and Systems, Northeast Agricultural University, Harbin, China; ^4^ College of Agriculture, Northeast Agricultural University, Harbin, China; ^5^ Grain Crops Institute, XinJiang Academy of Agricultural Sciences, Urumqi, China

**Keywords:** soybean, pod type, deep learning, metric learning, non-disassembled plants

## Abstract

Pod numbers are important for assessing soybean yield. How to simplify the traditional manual process and determine the pod number phenotype of soybean maturity more quickly and accurately is an urgent challenge for breeders. With the development of smart agriculture, numerous scientists have explored the phenotypic information related to soybean pod number and proposed corresponding methods. However, these methods mainly focus on the total number of pods, ignoring the differences between different pod types and do not consider the time-consuming and labor-intensive problem of picking pods from the whole plant. In this study, a deep learning approach was used to directly detect the number of different types of pods on non-disassembled plants at the maturity stage of soybean. Subsequently, the number of pods wascorrected by means of a metric learning method, thereby improving the accuracy of counting different types of pods. After 200 epochs, the recognition results of various object detection algorithms were compared to obtain the optimal model. Among the algorithms, YOLOX exhibited the highest mean average precision (mAP) of 83.43% in accurately determining the counts of diverse pod categories within soybean plants. By improving the Siamese Network in metric learning, the optimal Siamese Network model was obtained. SE-ResNet50 was used as the feature extraction network, and its accuracy on the test set reached 93.7%. Through the Siamese Network model, the results of object detection were further corrected and counted. The correlation coefficients between the number of one-seed pods, the number of two-seed pods, the number of three-seed pods, the number of four-seed pods and the total number of pods extracted by the algorithm and the manual measurement results were 92.62%, 95.17%, 96.90%, 94.93%, 96.64%,respectively. Compared with the object detection algorithm, the recognition of soybean mature pods was greatly improved, evolving into a high-throughput and universally applicable method. The described results show that the proposed method is a robust measurement and counting algorithm, which can reduce labor intensity, improve efficiency and accelerate the process of soybean breeding.

## 1 Introduction

Soybean, a prominent global agricultural commodity, holds a pivotal role as an essential provider of plant-based protein and oil for everyday human consumption. The phenotypic data associated with soybean holds relevance not only for yield estimation but also for quality assessment and other issues (Agarwal et al., 2013). In the field of soybean breeding, the thorough examination of soybean phenotypes is an essential endeavor. The relationship between soybean yield and quality can be explored by investigating the phenotypic characteristics, which is of considerable significance for breeding experts, since further research can be conducted based on the results. At present, the main focus of phenotypic research on soybean plants has been on the following aspects: pest detection, grain detection, yield estimation, maturity and others.

The quality of soybean seeds has a significant impact on the yield, and the problems of seed quality will affect the raw materials of agricultural comprehensive enterprises. Huang et al. (2022) designed a complete pipeline to classify soybean seeds, which was divided into segmentation and classification stages. The segmentation stage is performed by the popular deep learning method Mask R-CNN, and the classification stage is performed by a new network called Soybean Network (SNet). The proposed SNet model reportedly achieved a recognition accuracy of 96.2% with only 1.29M parameters. Park and Jun (2022) conducted phenotypic analysis of soybean seeds using artificial intelligence (AI) based on the YOLOv5 model. The mean average precision values of the model applied in the three soybean seed categories reached 0.835, 0.739 and 0.785. Li et al. (2021) proposed a model based on hyperspectral imaging technology and one-dimensional convolutional neural network (1D CNN) to distinguish soybean seed varieties, and the model achieved over 95% accuracy on both the training set and the validation set.

Anticipating crop yield prior to harvest is of considerable significance in shaping food policies and ensuring food security. Additionally, early prediction of yield at the field or plot level is beneficial for streamlined high-throughput plant phenotypic analysis and the implementation of precision agriculture practices. Maitiniyazi Maimaitijiang et al. (2020) developed a low-cost multi-sensor UAV for crop monitoring and phenotypic analysis. The data obtained by the UAV can relatively accurately and robustly estimate soybean (Glycine max) grain yield within the framework of deep neural networks (DNNs). A deep CNN-LSTM model was constructed by Sun et al. (2019) for county-level end-of-season and mid-season soybean yield prediction. The results revealed that the proposed CNN-LSTM model can achieve a favorable level of accuracy in both end-of-season and mid-season yield predictions. Riera et al. (2021) developed a multi-view image-based yield estimation framework using deep learning architecture. In the method, plant image fusion captured from different angles is used to estimate yield, and then soybean genotypes are sorted for breeding decisions.

Soybean plants exhibit a multitude of phenotypic traits, each possessing its own distinct research significance. Accurate and rapid extraction of soybean plant phenotypes has a guiding role in increasing yield and improving quality. Teodoro et al. (2021) proposed a machine learning method to predict the maturity days, plant height and grain yield of soybean varieties based on multi-spectral bands. The method mainly involves a multi-layer deep learning regression network, which can predict multiple important soybean crop phenotypes based on remote sensing data. At the same time, Moeinizade et al. (2022) used an end-to-end hybrid model combining convolutional neural networks and long-term and short-term memory. The model was used to extract features and capture the sequential behavior of time series data as a representation of soybean maturity, showing promise as an effective method. A new convolutional neural network architecture, named DS-SoybeanNet, was designed by Zhang et al. (2022) for improving the performance of soybean maturity information monitoring based on unmanned aerial vehicle (UAV) technology. Finally, the calculation speed of DS-SoybeanNet on every 1000 images was 11.770 seconds, and the F1 accuracy was 99.19%.

The swift advancement of deep learning is progressively delving into the exploration of soybean phenotypes, leading to the acquisition of an expanding array of phenotypic data. However, the existing research on soybean phenotype is mainly based on a macro perspective, such as the use of UAVs (Alabi et al., 2022; Yamamoto et al., 2023; Xu et al., 2023) and other equipment to estimate the overall yield. Such methods have exhibited low accuracy. Moreover, there are also several examples of exploration from the individual point of view, such as dismantling the whole soybean pods and grains for counting, but such methods require a large amount of manual participation, time and labor. In addition, the abundance of soybean phenotypes can sometimes lead to oversight of other crucial phenotypic details, such as the characteristics of soybean leaves and roots. As such, it is necessary to consider the diversity of soybean phenotypes more comprehensively and explore more reliable phenotypic information acquisition methods. Achieving high crop yields is the common standard and ultimate goal of most breeding programs, and thus requires large-scale yield assessments in soybean breeding (Guo et al., 2022). Since food estimation is usually assessed using labor-intensive methods only after harvest, an effective yield estimation method will greatly help breeders to focus on solving biological problems rather than being distracted by troublesome yield estimation models (Crusiol et al., 2022). The existing yield estimation methods predominantly concentrate on counting soybean pods and grains. However, such approach necessitates manual involvement to segregate pod grains from the entire plant. Unlike corn or wheat ears, soybean pods are densely arranged, resulting in pronounced pod overlap. Consequently, accurately identifying and localizing all pods within an image becomes a challenging task. In order to achieve accurate detection and counting, it is necessary to remove the pods from the branches to avoid overlap, so that the detection of pods can obtain a higher recognition accuracy. At the same time, the quantity of soybean pods inadequately represents soybean yield, as there exists a variety of pod types. These pod types encompass one-seed, two-seed, three-seed, and four-seed pods, with the possibility of mutant occurrences like five-seed or six-seed pods not being excluded.

The number of soybean pods is the most direct embodiment of soybean yield, and a large number of studies have been conducted on this issue. Li et al. (2019) first introduced a new large-scale seed counting dataset, which contained 500 annotated pod images with a total of 32,126 seeds. A dual-column Convolutional Neural Network (TCNN) technique was also formulated to elucidate pod images by transforming them into seed density maps. This process culminated in accurate seed counting, resulting in an average absolute error of 13.21 and a mean square error of 17.62. Yang et al. (2022) designed a two-step transfer learning method. In the first step, the instance segmentation network preprocessed by the source domain (MS COCO dataset) is fine-tuned with the synthetic object domain (*in vitro* soybean pod dataset). In the second step, by fine-tuning several real-world mature soybean plant samples, the conversion from simulation to reality can be achieved. The method was reported to achieve an AP value of 0.8. Uzal et al. (2018) introduced a computer vision method to distinguish different pod numbers. This method can estimate the number of seeds in soybean pods, and the final test accuracy rate reached 86.2%. Xiang et al. (2023) proposed a method based on the YOLOX framework called YOLO POD. The results showed that the R^2^ between the number predicted by YOLO POD and ground truth reached 0.967, which was 0.049 higher than that of YOLOX, while the inference time only increased by 0.08s. Further, MAE, MAPE and RMSE were only 4.18, 10.0% and 6.48, respectively, and the deviation was considerably small.

Through the continuous efforts of both domestic and international scholars, pod counting has been gradually improved, but there remains no classification and recognition of non-dismantled pods. The aim of most research has been on pod counting or dismantling pod classification and recognition, resulting in a vacancy in non-dismantled pod classification and recognition. Drawing from the intact nature of mature soybean plants, a new method was proposed in the present study. A new method was devised, harnessing the capabilities of deep learning and metric learning, to automatically and accurately identify mature soybean pods. Notably, the proposed method preserves the integrity of soybean plants without necessitating their destruction. The object detection method is first used to identify the pods in both the frontal and rear orientations of the same soybean plant, and then the similarities of the pods in the frontal and rear images are compared through the metric learning method to reduce the recognition error caused by occlusion. All the pods and the numbers of various types of pods in the non-dismantled soybean plant are then quickly and accurately detected. The method can measure and count quickly and accurately with high throughput, and does not destroy the overall morphological characteristics of soybean plants, avoiding the loss of phenotypic information caused by dismantling soybean plants.

## 2 Materials and methods

**Figure 1:**
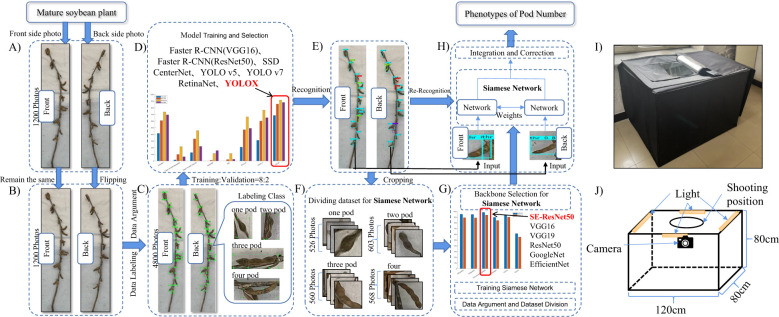
Research process. **(A)** Input image. **(B)** Mirror reflection. **(C)** Data labeling and augment. **(D)** Model train and select. **(E)** Recognition. **(F)** Crop images. **(G)** Improved Siamese Network. **(H)** Pod counting. **(I)** Object pictures. **(J)** Structural diagram.

An overview of the proposed method is given in **Figure 1**. The input of the system includes a series of images of different soybean varieties taken in a specific environment (using different planting dates and planting methods), in which the same soybean was photographed in both the frontal and rear orientations. Firstly, the collected image is preprocessed, and the reverse image of the same soybean plant is mirrored and flipped to match the positive image. According to the ratio of training set: verification set= 8: 2, the image is input into a variety of deep learning networks for detection and training optimization. By comparing the test results, the optimal network is selected as the method to detect the pods of soybean maturity. Then, the identified pods are cropped and re-divided into data sets as training samples for metric learning. The backbone network is replaced by the Siamese Network in metric learning and the model structure is improved to achieve the optimal results. Finally, the improved Siamese Network is used to accurately count the pods.

### 2.1 Image acquisition

In the present study, three kinds of soybeans with infinite podding habit, limited podding habit and sub-limited podding habit were selected as experimental materials. The experimental materials were planted in pots and fields in the experimental base of Northeast Agricultural University (45°361 N, 126°181 E) and Xiangyang Farm. A total of 350 soybean plants were harvested.

The RGB image acquisition platform was facilitated through an LED studio, depicted in **Figure 1**, possessing dimensions of 120cm x 80cm x 80cm. The exterior was made of black synthetic material and the interior was made of silver reflective material. Four LED light strips were installed on the top of the studio, which were distributed on the four frames at the top of the led studio, and reflective materials were provided around the studio to ensure sufficient lighting. There was a circular shooting port on the top, and the circular shooting port was filmed using an iPhone 13. In order to prevent the photo from being affected by background reflection, the background was composed of white light-absorbing cloth.

A dataset containing the pod counts of mature soybean plants was compiled. The images in the data set were all in JPG format and were taken by an iPhone 13. The direction of the shooting lens and the background cloth remained vertical to ensure clear shooting. To start, we positioned a soybean plant flat in the center of the LED studio, ensuring that the entire structure of the plant could be captured by a smartphone camera. Subsequently, an image of the soybean plant was taken using a smartphone. Once the initial capture was complete, the soybean plant was manually rotated 180 degrees to expose the portion that had been in contact with the ground. This allows you to capture images of the frontal and rear orientations of the soybean plant and then repeat the process with the same shot. The image resolution captured by iPhone 13 was 3024x4032.

At the same time of image acquisition, 50 soybean plants were selected as the reference plants to evaluate the performance of the algorithm. For the reference plants, the numbers of one-seed pods, two-seed pods, three-seed pods, and four-seed pods of each soybean were recorded. The present study was divided into two stages for the identification of soybean pods. The first stage was the classification and recognition stage of soybean pods, and 1200 soybean pod marker data sets were selected. The second stage was the pod correction counting stage. The data set used was the pods cut out after the prediction through the first stage, and each image contained a pod category.

### 2.2 Image preprocessing

For the processing of the object detection data set, the data set was marked with soybean pods by LabelImg software. After obtaining the soybean pod data set, the original data set was expanded by data enhancement technology, and a total of 4800 images were obtained. According to the ratio of training set: verification set = 8: 2, the images were input into a variety of deep learning networks for detection and training optimization.

For the processing of metric learning data sets, different types of pods were manually screened, and the selected one-seed pod, two-seed pod, three-seed pod, and four-seed pod images were placed under folders representing different pod numbers as data sets for training Siamese Networks. First, in order to improve the accuracy of the model and avoid the occurrence of over-fitting, techniques such as mirroring, adding salt and pepper noise, and rotating 180 degrees were applied to enhance the data set. The effects are shown in **Supplementary Figure S1**. The data set obtained by the method was input into the Siamese Network model for training.

### 2.3 Object detection

In order to obtain the optimal detection effect, a variety of object detection algorithms were selected for the selection scheme, namely a typical two-stage object detection algorithm, Faster Region Convolutional Neural Network (Ren et al., 2015), and training with Resnet50 (He et al., 2016) and VGG16 (Simonyan and Zisserman, 2014) as backbone networks; one-stage object detection algorithms, SSD (Liu et al., 2016), YOLO v5, YOLOX (Ge et al., 2021), YOLO v7 (Wang et al., 2023), RetinaNet (Lin et al., 2017) and CenterNet (Duan et al., 2019). Each model underwent 200 epochs of training, and the optimal outcomes were achieved by continuously optimizing the hyperparameters. CNN was trained on the soybean mature plant data set using the present authors’ computer hardware solution. The detailed experimental environment is shown in **Supplementary Table S1**.

### 2.4 Metric learning

The procedure involving the acquisition of knowledge from an input dataset to establish a technique for quantifying distinctions among various data entities is referred to as metric learning. For traditional metric learning, due to its limited ability to process raw data, the knowledge of feature engineering needs to be used first to preprocess the data, and then the metric learning algorithm needs to be used for learning. Certain traditional metric learning methods can only learn linear features. Although some kernel methods that can extract nonlinear features have been proposed, there was no significant improvement in the learning effect (Zhao et al., 2021; Kaya and Bilge, 2019; Hoffer and Ailon, 2015). With the emergence of deep learning, owing to the ability of the activation function to learn nonlinear features, deep learning methods can automatically learn high-quality features from the original data. As such, the combination of deep learning network structure and traditional metric learning methods can bring ideal results, and the Siamese Network (Chopra et al., 2005) is a typical metric learning model.

**Figure 2:**
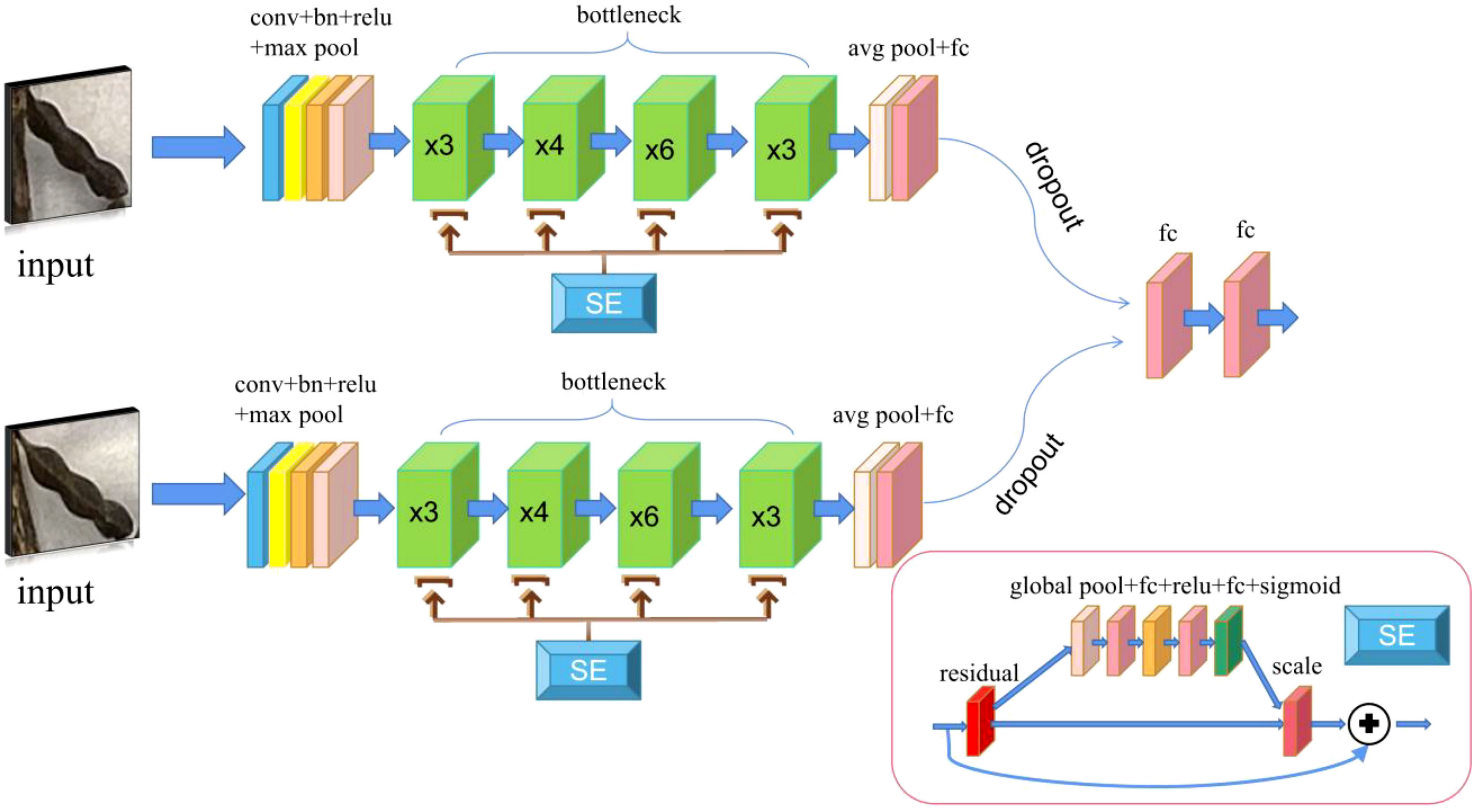
SE-Siamese Network. The two input images are processed through convolutional layers, batch normalization (BN) layers, and activation functions with identical structures, followed by a max pooling operation. The resulting features are then passed into a bottleneck module enhanced with a squeeze-and-excitation (SE) attention mechanism, and finally output through two fully connected layers.

In the present study, by comparing the Siamese Network models using different feature extraction networks, the optimal structure was selected for the similarity comparison of the two images. The commonly used Siamese Network uses VGG16 as the feature extraction network. In the present study, the performance of the network did not meet the study requirements. Therefore, the feature extraction network was substituted with alternative options, including ResNet50, GoogLeNet (Szegedy et al., 2015), EfficientNet (Tan and Le, 2019) and an improved version of ResNet50 (known as SE-ResNet50). The newly proposed Siamese Network improvement module is shown in **Figure 2**.

The new Siamese Network uses ResNet50 as the feature extraction network. Based on the original network model, the content of the residual block was changed, and the SE (Squeeze-and-Excitation) (Hu et al., 2018). module was added, that is, the SE attention mechanism. Through the SE attention mechanism, the features can be corrected, the valuable features can be retained, and the worthless features can be eliminated, thereby allowing the network to easily obtain more important feature information. After adding the SE attention mechanism module, the Dropout (Hinton et al., 2012) layer was also added to the network classification layer to avoid over-fitting issues. Through the aforementioned operations, a SE-Siamese Network was constructed.

### 2.5 Frontal and rear image correction method

The method mainly involves two stages. The first stage is the object detection stage. The pods of each category are identified and cut by the object detection method. At the same time, a folder is established according to the image name for unified preservation of the cut image. In the second stage, the similarities of all the detected pods of the frontal and rear images of the same soybean plant are compared through the Siamese Network. The highest similarity is considered to be the same soybean pod and recorded. When the similarity falls below the predefined threshold, it signifies that the soybean pod exclusively appears within a soybean image, and this occurrence is recorded simultaneously. Finally, the number of various types of pods in the statistical record is the pod phenotype information of the soybean. For instance, let’s consider the detection results from object detection on both sides of a soybean plant. We first select a pod $f_{a}$ as the target pod in the frontal soybean pod set $f=\{f_{1},f_{2},\ldots ,f_{n}\}$ . Simultaneously, we iterate through all rear pod sets $p=\{p_{1},p_{2},\ldots ,p_{n}\}$ to find the most similar pod $p_{m}$ in set *p* that matches the target $f_{a}$ . Upon finding a match, $f_{a}$ and $p_{m}$ are considered the same pod, and both are then removed from sets *f* and *p*, respectively. The hyperparameter threshold is set to 0.5, indicating that if there exists a pod in set *p* with a similarity score greater than 0.5 to the target pod $f_{a}$ , it is also considered the same pod. Otherwise, the target pod $f_{a}$ is considered to exist only on one side, so the identified pod $f_{a}$ is recorded, and its corresponding pod number is removed from set *f*. This process iterates through set *f*, enabling the counting of various pods of a soybean plant while considering occlusions and duplications across both sides of the plant. As illustrated in **Supplementary Figure S2**, when one side of the pod is missed while the other side is successfully detected, this method can be employed to perform supplementary pod counting.

### 2.6 Evaluation index and statistical analysis

The results of different networks used on the dataset were evaluated. For an evaluation, if the detected instance has a Jaccard index similarity coefficient, also known as the intersection (IOU), it is considered to be a true positive (Csurka et al., 2013; He and Garcia, 2009) of 0.5 or higher, with a basic fact instance. IOU is defined as the ratio of the number of pixels in the intersection set to the number of pixels in the union set. Ground live instances that do not overlap with any detected instances are considered false negatives. According to the described measures, the Precision , Recall , F1 (Afonso et al., 2020), AP and mAP can be calculated as follows Equations 1–5:


(1) $$Precision=\frac{TP}{TP+FP}$$

(2) $$Recall=\frac{TP}{TP+FN}$$

(3) $$F1=\frac{2Precision\times Recall}{Precision+Recall}$$

(4) $$AP=\sum_{k=1}^{N}Precision(k)\Delta Recall(k)$$

(5) $$mAP=\frac{\sum_{i}^{M}AP_{i}}{M}$$

where TP = the number of true positives; FP = the number of false positives; FN = the number of false negatives; *N* is the total number of images in the test dataset; *M* is the number of classes; Precision(k) is the precision value at images; and ΔRecall(k) is the recall change between the *k* and k−1 images.

Further, the mean absolute error ( MAE ), root mean squared error ( RMSE ), and the correlation coefficient (*R*) were used as the evaluation metrics to assess the counting performance. The three metrics can be denoted as follows: They take the forms Equations 6–8:


(6) $$MAE=\frac{1}{N}\sum_{i=1}^{N}\left|t_{i}-c_{i}\right|$$

(7) $$RMSE=\sqrt{\frac{1}{N}\sum_{i=1}^{N}{\left(t_{i}-c_{i}\right)}^{2}}$$

(8) $$R=\sqrt{1-\frac{\sum_{i=1}^{N}{\left(t_{i}-c_{i}\right)}^{2}}{\sum_{i=1}^{N}{\left(t_{i}-\bar{t}\right)}^{2}}}$$

where *N* denotes the number of test images; $t_{i}$ is the ground truth count for the i−th image; $c_{i}$ is the inferred count for the i−th image; and $\bar{t}$ is the mean of $t_{i}$ .

In addition, an evaluation was first conducted with regard to the accuracy of soybean pod classification and counting using only the object detection algorithm and the fusion algorithm based on object detection and metric learning. The calculation formula is as follows Equation 9:


(9) $$Acc=1-\frac{\left|truth-predict\right|}{truth}$$

Where Acc represents the accuracy index obtained by detection; truth represents the ground truth of the number of pods obtained by manual counting; and predict represents the prediction of the number of pods obtained by algorithm counting.

In order to evaluate the role of the object detection algorithm in the actual pod classification and counting, different models were used in the object detection stage to evaluate the final pod classification and counting performance. Additionally, the average accuracy was used to measure the performance of each fusion algorithm. The formula is as follows Equation 10:


(10) $$Acc_{mean}=\frac{SUM(Acc_{i})}{5},i=1,2,\ldots ,5$$

The formula $Acc_{mean}$ represents the average accuracy of each object detection algorithm in all categories, SUM represents the summation function, and $Acc_{i}$ represents the accuracy of each category. The five categories here refer to one-seed pods, two-seed pods, three-seed pods, four-seed pods and total pods.

### 2.7 Experimental settings

The present experiment was implemented using Pytorch 1.10.1. Firstly, the selected object detection network was trained. In the training stage, different optimizers were used to update each network according to different network characteristics. At the same time, in different object detection models, different initial learning rates were used to increase the speed of the network convergence. In the experiment, each network was subjected to iterative training over 200 epochs, resulting in successful convergence. The initial value of momentum was selected according to the characteristics of each network, and the detailed information is shown in **Supplementary Table S2**.

## 3 Results

### 3.1 Training and evaluation of object detection model for pods

**Figure 3:**
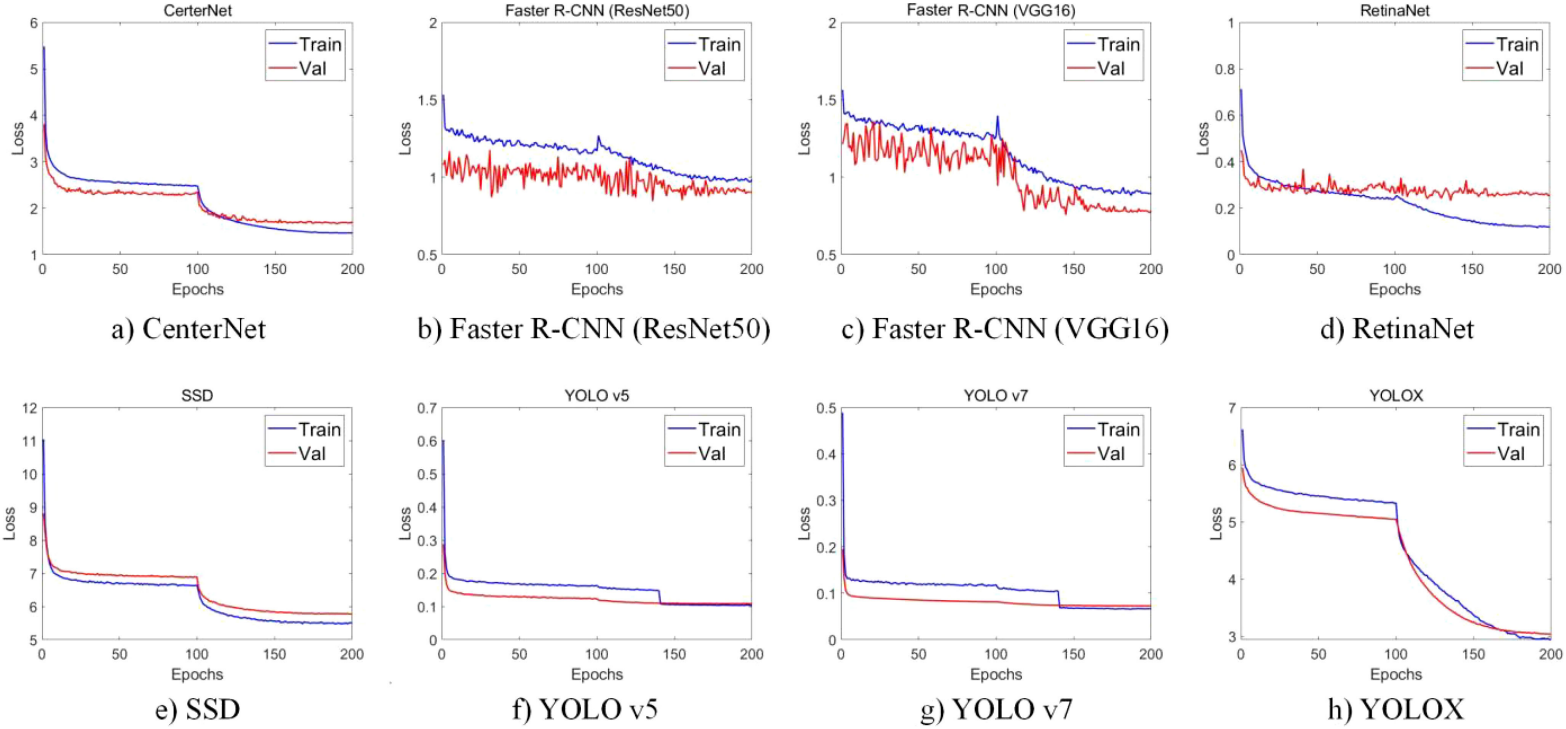
Change curve of loss function in training and verification process. **(a)** CenterNet. **(b)** Faster R-CNN (ResNet50). **(c)** Faster R-CNN (VGG16). **(d)** RetinaNet. **(e)** SSD. **(f)** YOLO v5. **(g)** YOLO v7. **(h)** YOLOX.

The first stage involved using the object detection algorithm to find the optimal CNN model for different types of soybean pod detection. In the present study, YOLOX, YOLO v5, YOLO v7, SSD, CenterNet, RetinaNet and Faster R-CNN (VGG16), Faster R-CNN (ResNet50) were trained and evaluated. After 200 epochs of training, the convergence of the network was analyzed. The loss function curve of the training and verification process is shown in **Figure 3**. An observation can be made that Faster R-CNN (VGG16), Faster R-CNN (ResNet50) and RetinaNet oscillated more severely during the training process, which may be caused by the excessive learning rate and the inability to find the optimal solution. At the beginning of the training phase, the training loss of the remaining networks decreased sharply, and then after a certain number of iterations, the loss value slowly converged to an accurate value. Such findings could be attributed to the model not being accurate enough in the early stage of network training, and more iterations being needed to make the model gradually converge to an accurate state.

After the convergence of the model, the test set data of each model was analyzed, and the evaluation indexes such as mAP , AP , Precision , Recall , F1 and FPS on the test set were obtained, as shown in **Table 1**. Through comparing the performance of several models, an observation can be made that the FPS speed of SSD was the fastest, reaching 154.97, but its mAP was only 3.97%, and the detection effect for the pod category was poor. Regarding mAP, YOLOX exhibited the optimal performance across all models, reaching 83.43%, but FPS was much lower than SSD at only 37.26. Across all the models, it is evident that, with the exception of the two-stage object detection algorithm Faster R-CNN, the Precision values of the remaining models surpassed their Recall values. This observation suggests that the one-stage object detection algorithm is proficient in generating accurate prediction outcomes. However, its capability to accurately identify real results is comparatively limited. A further observation can be made that the recognition accuracy of three-seed pods was the highest among all pod categories. This phenomenon can be attributed to two factors. Firstly, the abundance of samples contributes to this outcome, as three-seed pods constituted the largest subset among all pods. Secondly, the distinct attributes of three-seed pods might also have contributed to this heightened accuracy. Conversely, the recognition effect of one pod was the lowest. This outcome is attributed, firstly, to the limited quantity of available samples, and secondly, to the inherent traits of single-seed pods. These traits include their diminutive size, susceptibility to occlusion, and challenging detectability.

**Table 1:** Performance of different networks.

Network name	Category	Precision	Recall	F1	AP	mAP	FPS
Faster R-CNN (ResNet50)	one two three four	10.75% 23.62% 29.81% 14.94%	3.18% 12.57% 35.56% 16.98%	0.05 0.16 0.32 0.16	1.74% 9.90% 21.55% 6.38%	9.89%	12.37
Faster R-CNN (VGG16)	one two three four	26.60% 36.54% 45.29% 27.33%	18.38% 41.92% 59.68% 39.02%	0.22 0.39 0.51 0.32	12.72% 33.20% 45.59% 21.92%	28.36%	11.77
SSD	one two three four	0.01% 0.01% 30% 13.33%	0.01% 0.01% 0.05% 0.15%	0.01 0.01 0.01 0.01	0.01% 2.16% 11.25% 2.48%	3.97%	154.97
CenterNet	one two three four	85.21% 90.92% 91.78% 85.21%	8.36% 17.66% 34.21% 42.72%	0.15 0.30 0.50 0.58	41.66% 60.81% 73.71% 69.36%	61.38%	86.89
RetinaNet	one two three four	100.00% 76.92% 60.23% 69.66%	0.07% 0.22% 1.62% 4.68%	0.00 0.00 0.03 0.09	0.07% 1.15% 11.39% 15.68%	7.07%	45.93
YOLO v5	one two three four	94.87% 81.62% 76.18% 78.65%	2.56% 9.19% 41.49% 39.47%	0.05 0.17 0.54 0.53	20.90% 47.08% 66.46% 49.07%	45.87%	36.29
YOLO v7	one two three four	92.86% 80.79% 80.13% 79.11%	3.59% 26.20% 58.59% 54.87%	0.07 0.40 0.68 0.65	31.19% 60.17% 76.61% 65.18%	58.29%	47.16
YOLOX	one two three four	85.23% 88.69% 90.94% 89.54%	59.43% 77.05% 86.79% 85.28%	0.70 0.82 0.89 0.87	68.43% 85.66% 91.55% 88.09%	83.43%	37.26

In addition, in order to intuitively show the different prediction effects of different network models, the prediction results of different networks were obtained, as shown in **Supplementary Figure S2**. The evaluation results demonstrate that, when compared to the other networks, SSD exhibited the least favorable predictive capability, failing to accurately capture conspicuous pod information and essentially yielding negligible performance. RetinaNet, while somewhat improved, was still inadequate in detecting only a portion of the three-seed pods, unable to identify other pod types. The two renowned two-stage object detection networks, Faster R-CNN (VGG16) and Faster R-CNN (ResNet50), demonstrated notably enhanced performance, yet they did not achieve the desired level. In contrast, the YOLO series network and CenterNet exhibited the most effective detection performance. Notably, YOLOX had the highest recognition accuracy at 83.43%. After considering multiple factors, YOLOX was adopted as the chosen network for one-stage object detection.

### 3.2 Metric learning model training and evaluation

The Siamese Network performances of VGG16, VGG19, ResNet50, EfficientNet, GoogLeNet and SE-ResNet50 as different feature extraction networks were compared. At the same time, for the selected optimal model, the optimal accuracy was explored by adjusting the hyperparameters for further detailed analysis.

For different feature extraction networks, the following hyperparameter settings were adopted. The image input dimensions were configured as 105x105, with the network undergoing training for 200 epochs. The batch size was established at 32, and the initial learning rate was set to 1e-2. The training process employed SGD with a momentum value of 0.9. Meanwhile, in order to avoid the problems associated with slow training from initial conditions and the suboptimal training outcomes stemming from random weight initialization, transfer learning (Pan and Yang, 2009; Zhuang et al., 2020) was used to pretrain the model, and the accuracy of each feature extraction network was obtained as shown in **Table 2**.

**Table 2:** Comparison of training and test values of different feature extraction networks.

Models	Train(%)	Test(%)
Siamese Network(VGG16)	93.4	90.1
Siamese Network(VGG19)	61.2	55.4
Siamese Network(ResNet50)	89.7	84.2
Siamese Network(EfficientNet)	95.1	89.2
Siamese Network(GoogLeNet)	94.7	88.7
Siamese Network(SE-ResNet50)	98.4	93.7

The experimental results show that the SE-ResNet50 model as the feature extraction network of the Siamese Network achieved the highest training accuracy of 98.4%, and also had the highest test accuracy of 93.7%. Compared with the original VGG16 as the feature extraction network, the training accuracy of the Siamese Network model was improved by 5%, and the accuracy of the test set was improved by 3.6%. Compared with the unimproved ResNet50 as the feature extraction network, the training accuracy of the Siamese Network was improved by 8.7%, and the test accuracy was improved by 9.5%. Among all the models, the Siamese Network model with VGG19 as the feature extraction network had the lowest training accuracy, with an accuracy of only 61.2%. The Siamese Network model with VGG19 as the feature extraction network still had the lowest test accuracy, with an accuracy of 55.4%.

**Figure 4:**
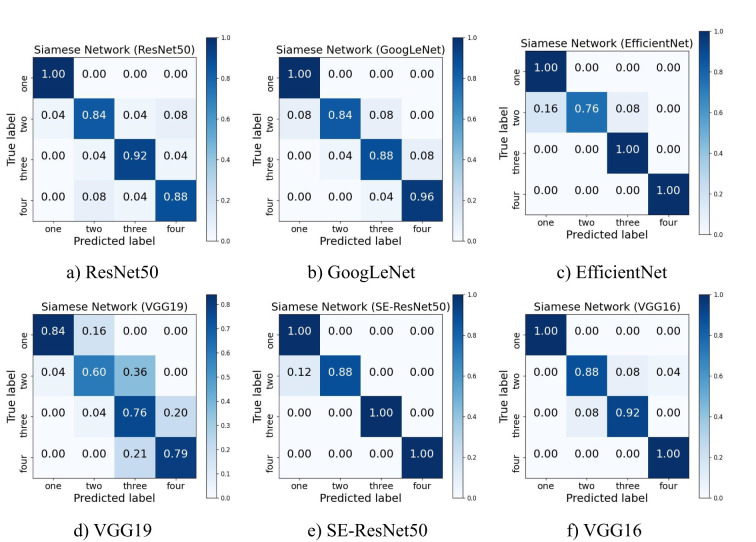
Confusion matrix of six feature extraction networks. **(a)** ResNet50. **(b)** GoogLeNet. **(s)** EfficientNet. **(d)** VGG19. **(e)** SE-ResNet50. **(f)** VGG16.

In order to more intuitively show the prediction effect of each feature extraction network on soybean pods, several images of each type of pod were selected for prediction analysis from 50 soybean samples recorded in advance, and the confusion matrix shown in **Figure 4** was drawn. The ordinate represents the real one-seed pods, two-seed pods, three-seed pods and four-seed pods, and the abscissa represents the predicted one-seed pods, two-seed pods, three-seed pods and four-seed pods.

Through the observation of the confusion matrix, an observation can be made that in the judgment of all types of pods, the judgment accuracy of one-seed pods was the highest. Except for the Siamese Network model with VGG19 as the backbone feature extraction network, the judgment accuracy of one-seed pods in all other models could reach 100%. This outcome suggests a substantial distinction between one-seed pods and other pod types, enabling the network to accurately differentiate the distinct attributes of one-seed pods. On the contrary, the recognition accuracy of two-seed pods was the lowest among all types of pods, was more likely to be misjudged as a one-seed pod or three-seed pod. The only error in the Siamese Network model with SE-ResNet50 as the feature extraction network was the recognition of two-seed pods. Among all types of pods, the accuracy of four-seed pods was second only to that of one-seed pods. Such findings can be attributed to both one-seed pods and four-seed pods belonging to marginal pods. In essence, the entity most akin to a one-seed pod could be interpreted as a two-seed pod, and the entity most resembling a four-seed pod could be recognized as a three-seed pod. However, the relationship between two-seed pods and three-seed pods is less straightforward, rendering their characteristics intricate to capture, which consequently results in a lower recognition accuracy.

In order to achieve the optimal effect of the model, some hyperparameters in the network structure needed to be adjusted. In the experiment, the SE-Siamese Network model was trained with different hyperparameters to obtain different accuracy rates. The results are shown in **Supplementary Table S3**. An observation can be made that the highest accuracy of 93.1% was obtained in the case of optimal hyper-parameter combination (learning rate of 1e-3, momentum value of 0.9, batch size of 32). In general, the effect of using the Adam (Kingma, 2014) optimizer was generally higher than that of using the SGD optimizer, and different batch sizes had minimal effect on the overall accuracy. As is well known there is no fixed template for the setting of hyperparameters in CNN training. When setting, the characteristics of data sets and computing resources also need to be considered. The hyperparameters should not be blindly increased or decreased.

To summarize, the process of determining an optimal model involved evaluating network models utilizing diverse strategies. SE-ResNet50 was chosen as the backbone feature extraction network for the Siamese Network. A dropout rate of 0.2 was selected, along with a batch size of 32. The highest accuracy was attained when utilizing the Adam optimizer with a learning rate of 1e-3. To provide an intuitive insight into the image transformation process within the model, the visualization of the feature extraction component was performed, as depicted in **Supplementary Figure S3**.

From **Supplementary Figure S3**, an observation can be made that the results were obtained after multiple convolutions in the deep learning architecture. The results of multiple feature changes of one-seed pods, two-seed pods, three-seed pods and four-seed pods are shown. In the initial feature transformation, the structural features of various types of pods could be clearly extracted. Especially in the first two feature transformations, there appears to be no significant difference from the original image. However, with the deepening of the network and the increase in the number of convolutions, the image gradually became distorted and difficult to understand clearly after the third feature change. Overall, the feature extraction network could clearly extract the features of different types of pods.

### 3.3 Comparative analysis of experimental results

In the present study, the two-stage method of object detection and metric learning was used to give the recognition and counting method of different types of soybean pods, thereby allowing for the counting error caused by soybean pod occlusion to be effectively solved. As a one-stage processing method, the object detection method first detects and identifies different types of pods, and then automatically cuts the recognition object to input into the two-stage metric learning algorithm for similarity comparison. While the method’s efficiency is comparatively lower than that achieved by solely employing the object detection algorithm for soybean pod recognition, the trade-off is acceptable due to the algorithm’s enhanced accuracy. This warrants a marginal increase in computation time for the sake of accuracy improvement. In addition, for complex types of soybean plants, pods are dense, and the correction count of front and back images alone is not enough to fully obtain all pod information. At this time, the number of different types of pods of the whole soybean can be estimated by post-processing and other methods to achieve the result of approximating the ground truth.

**Figure 5:**
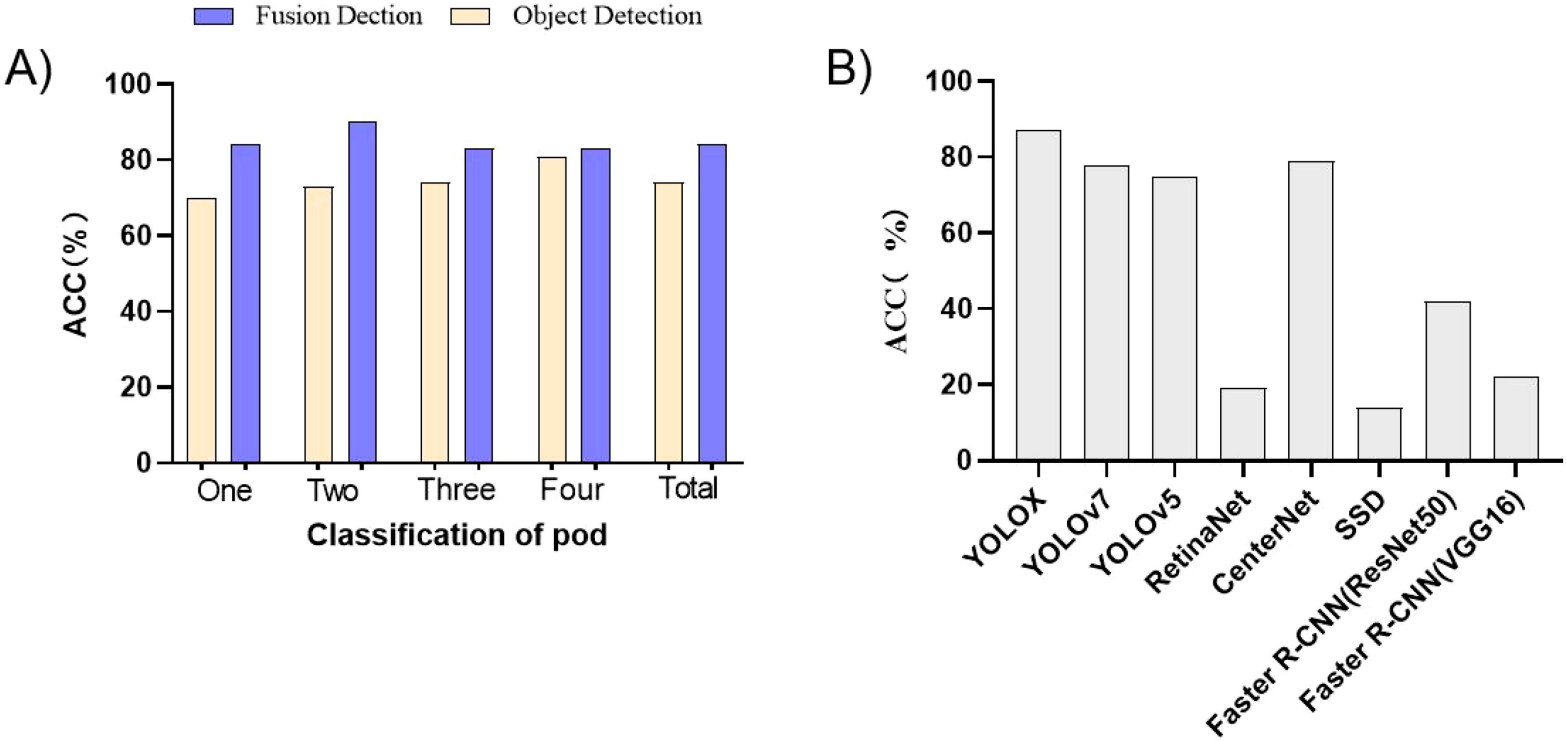
Comparative analysis. **(A)** Comparison of the accuracy of the object detection algorithm and the fusion algorithm. **(B)** Performance comparison of different object detection algorithms in actual pod classification and counting.

**Figure 5A** shows the accuracy comparison between the object detection algorithm and the fusion algorithm based on deep learning and metric learning. A clear observation can be made that the accuracy of the fusion algorithm based on deep learning and metric learning was higher than that of the object detection algorithm alone, whether on different categories of pods or for the total number of pods. Notably, the enhancement was more pronounced for one-seed pods and two-seed pods, whereas the improvement for four-seed pods was relatively marginal. The underlying cause can be attributed to the following: one-seed pods and two-seed pods possess a shorter length and are frequently concealed by other pods during the growth and development of soybeans. This obstructs their visibility from certain angles, whereas the four-seed pod, owing to its elongated structure, is less prone to being obstructed by neighboring pods. As such, the fusion algorithm brings about a more substantial enhancement in the recognition of one-seed and two-seed pods, while the improvement in the recognition of four-seed pods is relatively subdued.

From **Figure 5B**, an observation can be made that the accuracy of the object detection effect had a significant effect on the fusion model. The higher the accuracy of the object detection model, the better the final fusion model. Therefore, if the accuracy of the object detection algorithm can be improved, it will have a positive effect on the final improvement of the fusion model.

In order to clearly show that the accuracy of the fusion algorithm based on object detection and metric learning is higher than that of the object detection algorithm alone, several soybean plant images were selected from the test set, and the evaluation results of the detection using the object detection algorithm alone were compared with the evaluation results of the two-stage fusion algorithm, as shown in **Table 3**.

**Table 3:** Recognition performance of different models for soybean pods.

Seeds per pod	Object detection			Fusion algorithm		
	R	MAE	RMSE	R	MAE	RMSE
one	0.8547	0.8333	1.6330	0.9265	0.8167	1.2042
two	0.8890	2.4333	3.5355	0.9517	2.0500	2.5528
three	0.9105	4.4833	6.0539	0.9690	2.7500	3.2939
four	0.9093	1.3500	2.2023	0.9493	2.1500	2.7779
total	0.8856	8.0333	10.2794	0.9664	7.2000	7.7567

From the experimental results in **Table 3**, an observation can be made that the proposed fusion algorithm based on deep learning and metric learning was higher than the result of using the object detection algorithm alone in the correlation comparison. The correlation between the ground truth and the prediction of the three-seed pods reached the highest value of 0.9690, which was 0.0585 higher than that of the object detection algorithm alone. In addition, for the fusion algorithm based on object detection and metric learning, the correlation between the ground truth and the prediction was greater than 0.9, indicating that there was a high correlation between the predicted result and the ground truth. In the comparison of the root mean square error of **Table 3** and the average absolute error of **Table 3**, except for the error of the four-seed pods fusion model based on object detection and metric learning being higher than that of the model using only object detection, the fusion algorithm based on object detection and metric learning could achieve better results in the remaining errors. Overall, the selected soybean plant samples based on the fusion method of object detection and metric learning obviously exhibited clear advantages in terms of recognition accuracy.

## 4 Discussion

### 4.1 The bias of the number of pods in each category may be one of the reasons that affect the object detection results

In the present study, during the sampling process of soybean plants under natural conditions, the number of pods in each category was not the same, and the data samples obtained were divided in detail as shown in **Table 4**. From the statistical results, an observation can be made that the numbers of two-seed pods and three-seed pods in the obtained 1200 original images were far more than the numbers of one-seed pods and four-seed pods. Therefore, more pods with different shapes, textures and colors can be obtained in the feature extraction process for two-seed pods and three-seed pods, which is also one of the reasons for the high recognition accuracy of two-seed pods and three-seed pods in the final recognition process. Moreover, Yang et al. (2022) also explained the relationship between recognition results and data balance. Therefore, in order to obtain higher recognition accuracy, it is crucial to expand and balance the data of each category.

**Table 4:** Pod number statistics and object detection and recognition accuracy of each category.

Methods	One	Two	Three	Four
Number	3078	9805	13896	2955
Faster R-CNN (ResNet50)	1.74%	9.90%	21.55%	6.38%
Faster R-CNN (VGG16)	12.72%	33.20%	45.59%	21.92%
SSD	0.01%	2.16%	11.25%	2.48%
CenterNet	41.66%	60.81%	73.71%	69.36%
RetinaNet	0.07%	1.15%	11.39%	15.68%
YOLO v5	20.90%	47.08%	66.46%	49.07%
YOLO v7	31.19%	60.17%	76.61%	65.18%
YOLOX	68.43%	85.66%	91.55%	88.09%

### 4.2 Plant complexity is an important reason that affects the recognition effect of the model

**Figure 6:**
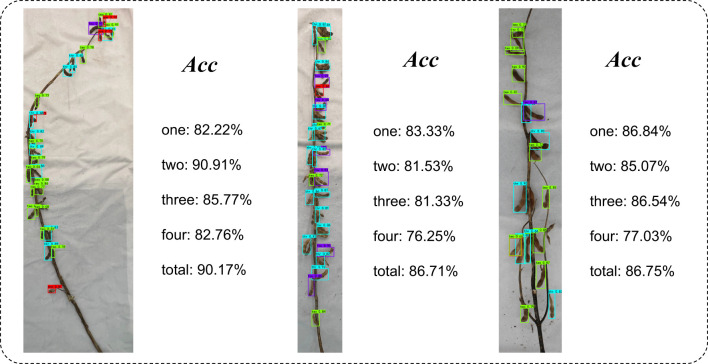
Comparison of phenotypic extraction accuracy of plants with different podding habits. **(A)** Infinite podding habit. **(B)** Sub-finite podding habit. **(C)** Finite podding habit.

The varying complexity of soybean plants significantly influences the ultimate recognition outcomes. In the present study, plants displaying infinite podding habits, sub-finite podding habits, and limited podding habits were carefully selected to thoroughly investigate and analyze recognition accuracy. Visual representation is shown in **Figure 6**. According to the results, the average accuracy of infinite podding habit pods was higher than that of sub-finite podding habit and finite podding habit pods. Such findings could be related to the characteristics of infinite podding habit, wherein the plant height is higher, the pitch is longer, and the overall number of pods is fewer. As a result, the final results were seldom marred by counting errors attributed to occlusion or similar factors. Notably, the shorter inter-pod spacing and higher pod density of plants with sub-finite podding habits and finite podding habits predominantly contribute to their lower recognition accuracy. In addition, the accuracy of the total pod number was slightly higher than the average accuracy. The reason for such findings is that some pod number prediction results exceeded the ground truth, while some pod number prediction results were lower than the ground truth, resulting in the total pod number being closer to the ground truth.

### 4.3 The total number of particles cannot be directly calculated by the calculated results

The total number of grains is the most accurate reflection of the yield of a soybean. The more the total number of grains of a soybean, the higher the yield of the soybean. The soybean will also become the focus of breeding experts. However, the total number of seeds cannot be calculated only by counting the sum of various types of pods on each soybean plant, because there are empty pods during the growth and development of soybeans, resulting in a slight error between the sum of different types of pods and the real number of soybean plants. Hence, the computation of the overall seed count in soybean plants remains a work in progress, necessitating further efforts to identify and address issues associated with empty pods (Uzal et al., 2018).

### 4.4 Impact of data imbalance on metric learning

While our Siamese Network achieves strong performance overall, we observe that data imbalance among pod types poses notable challenges during the metric learning stage. In particular, classes with fewer training samples tend to suffer from higher intra-class variance and lower inter-class separability, which in turn hinders the network’s ability to learn discriminative and robust feature embeddings for these underrepresented types. This effect is especially pronounced in pairwise similarity evaluation, where rare pod types exhibit higher rates of false negatives. The imbalance limits the diversity and informativeness of sample pairs involving low-frequency classes, leading the model to overfit to majority classes and potentially ignore minority-specific representations. As a result, the learned embedding space may not preserve sufficient semantic structure for rare classes, which impacts both retrieval-based and verification-style tasks. In future research, we plan to adopt a class-balanced sampling strategy to ensure that each training batch provides a more even distribution of pod types and prevent the majority class from dominating.

### 4.5 Some limitations

While our proposed fusion algorithm has demonstrated its effectiveness in the majority of experiments, it still exhibits certain limitations. Specifically, occlusions present in soybean plants when photographed from both directions can lead to errors in the object detection method, consequently impacting the performance of the metric learning method, which is closely intertwined with the effectiveness of object detection. Thus, the optimal approach in such scenarios would involve minimizing occlusion-induced errors through human intervention(for example, Manually expose the occluded pods so that the camera can capture them). Additionally, the performance of the object detection method significantly influences the final outcome. While our method offers some correction capabilities for counting, further enhancement of the object detection method would lead to improved pod counting results. This direction constitutes our next research endeavor.

## 5 Conclusions

In the present study, a high-precision automatic identification method of whole plant pods in soybean maturity based on deep learning and metric learning was proposed, which was elaborated from data acquisition, model construction and experimental results and analysis. With the aim of achieving recognition of different types of soybean pods, different object detection algorithms were used for recognition. The optimal model was selected as the detection model of the first stage, and its accuracy on the test set reached 83.43%. In the second stage, the Siamese Network models with different feature extraction networks were compared. According to the comparison results, the Siamese Network with SE-ResNet50 as the feature extraction network was the optimal model, and its accuracy on the test set reached 93.7%. Further, the hyperparameters of the optimal model SE-Siamese Network were compared, and the optimal combination model was obtained. Finally, the performance of the model was verified again by using the method of confusion matrix and feature map visualization. In order to demonstrate the effect of the model in practical application, the images of some soybean plants were selected, and the phenotypic information of different types of pods, total pods and total grains were manually counted. The correlation between the fusion algorithm based on deep learning and metric learning and the method using only object detection was compared. Findings were made that the correlation of the fusion method based on deep learning and metric learning was higher than that of the method using only object detection, which verifies the accuracy of the method and provides a new direction for other similar studies.

## Data Availability

The raw data supporting the conclusions of this article will be made available by the authors, without undue reservation.

## Supplementary material

Supplementary Figure 1 Results of different pod numbers using different data augmentation methods.

Supplementary Figure 2 A note on supplementary counting through metric learning.

Supplementary Figure 3 The prediction effect of different networks. **(A)**YOLOX. **(B)**YOLO v7. **(C)** YOLO v5. **(D)** Faster R-CNN(VGG16). **(E)** RetinaNet. **(F)** Faster R-CNN(ResNet50). **(G)** CenterNet. **(H)** SSD.

Supplementary Figure 4 The transformation process of different types of pods through feature extraction network.

Supplementary Table 1 Experimental environment settings.

Supplementary Table 2 Experimental parameter settings.

Supplementary Table 3 Effects of different hyperparameters on accuracy.
