# Supplementary figures and images for: DLML-PC: an automated deep learning and metric learning approach for precise soybean pod classification and counting in intact plants
Source: Front Plant Sci. 2025 Jul 21;16:1583526. doi: 10.3389/fpls.2025.1583526 (PMC12319039; doi:10.3389/fpls.2025.1583526)

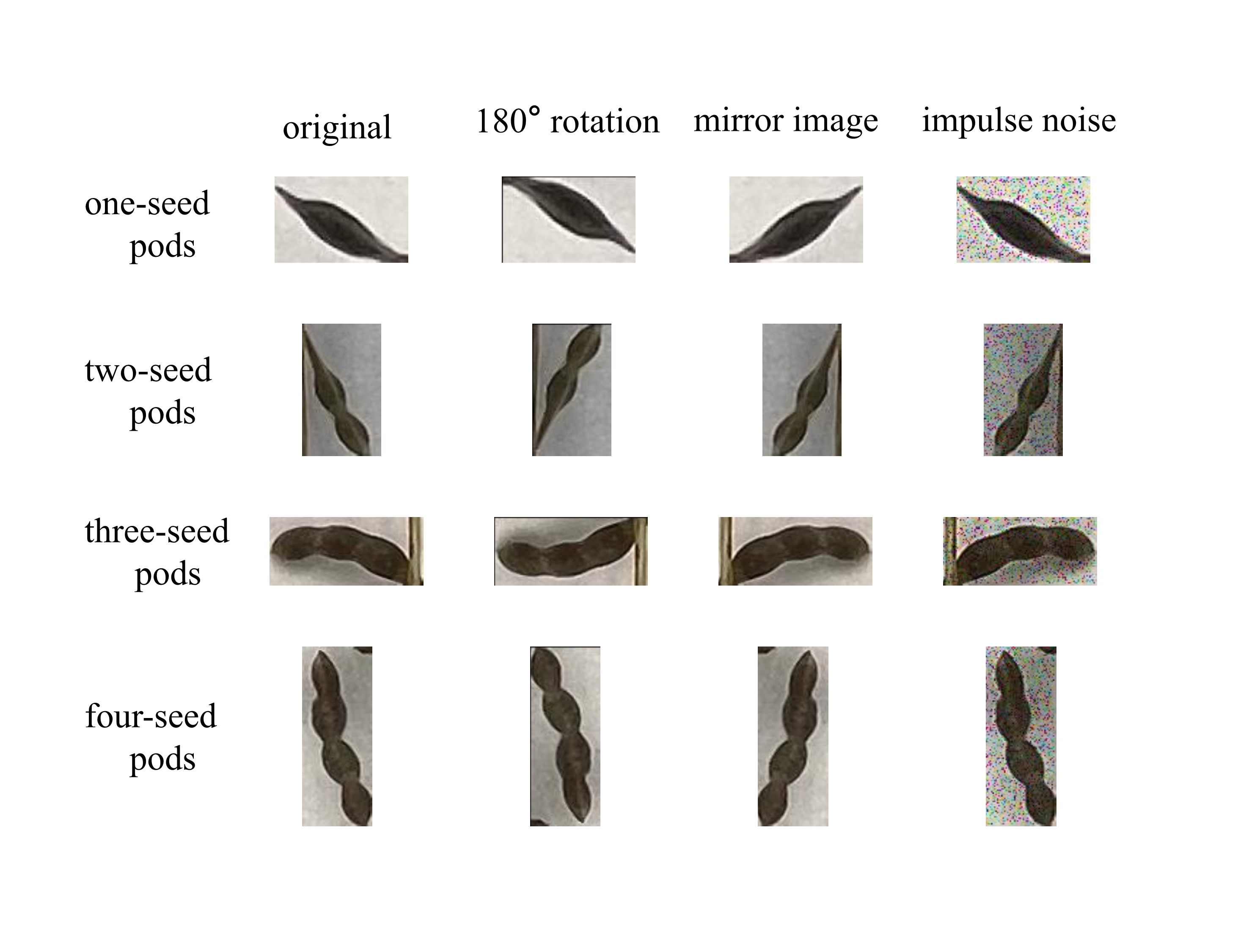

Supplement: Supplementary Figure 1 — Results of different pod numbers using different data augmentation methods. [file Image1.tiff]

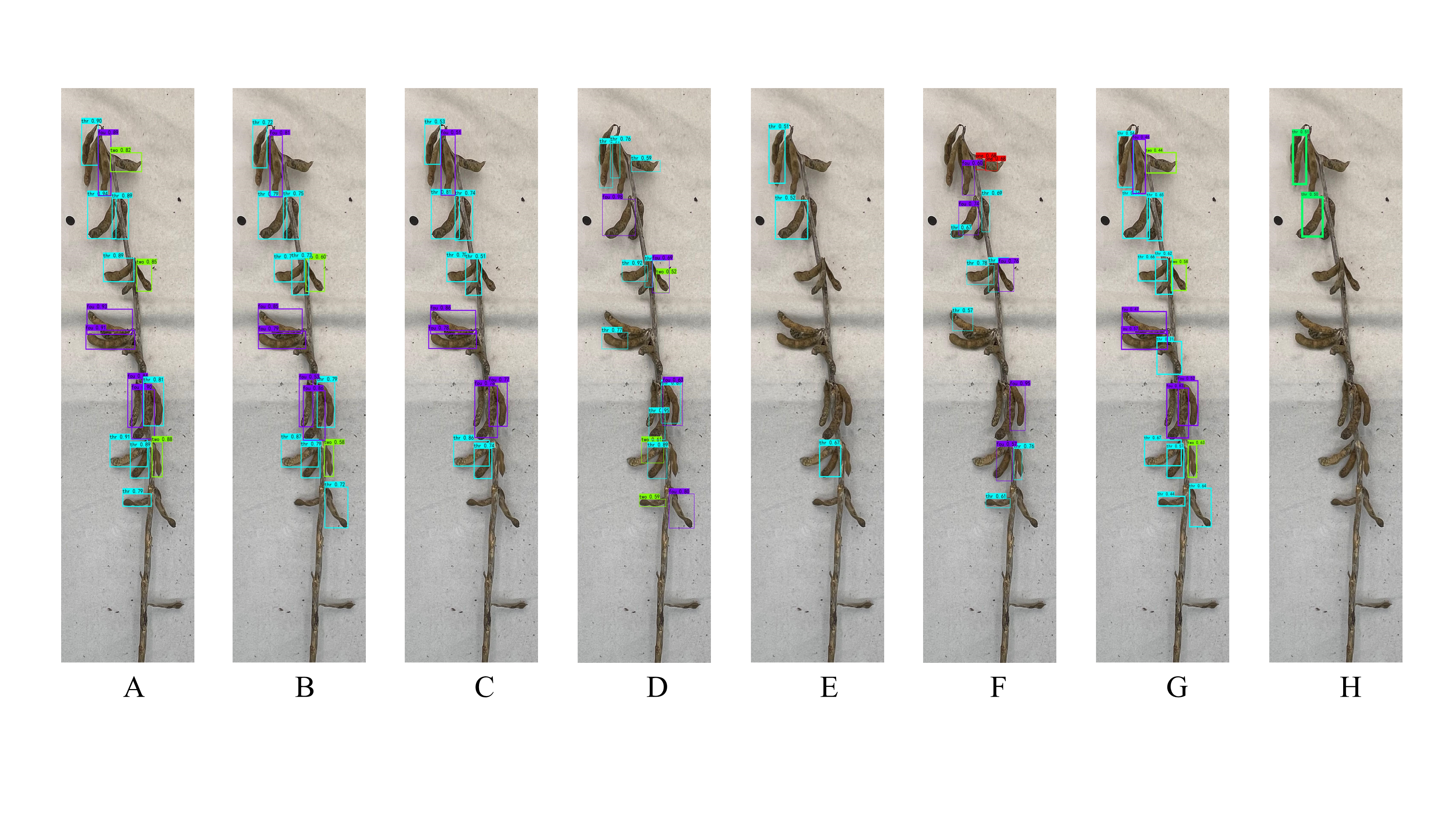

Supplement: Supplementary Figure 3 — The prediction effect of different networks. (A)YOLOX. (B)YOLO v7. (C) YOLO v5. (D) Faster R-CNN(VGG16). (E) RetinaNet. (F) Faster R-CNN(ResNet50). (G) CenterNet. (H) SSD. [file Image3.tiff]

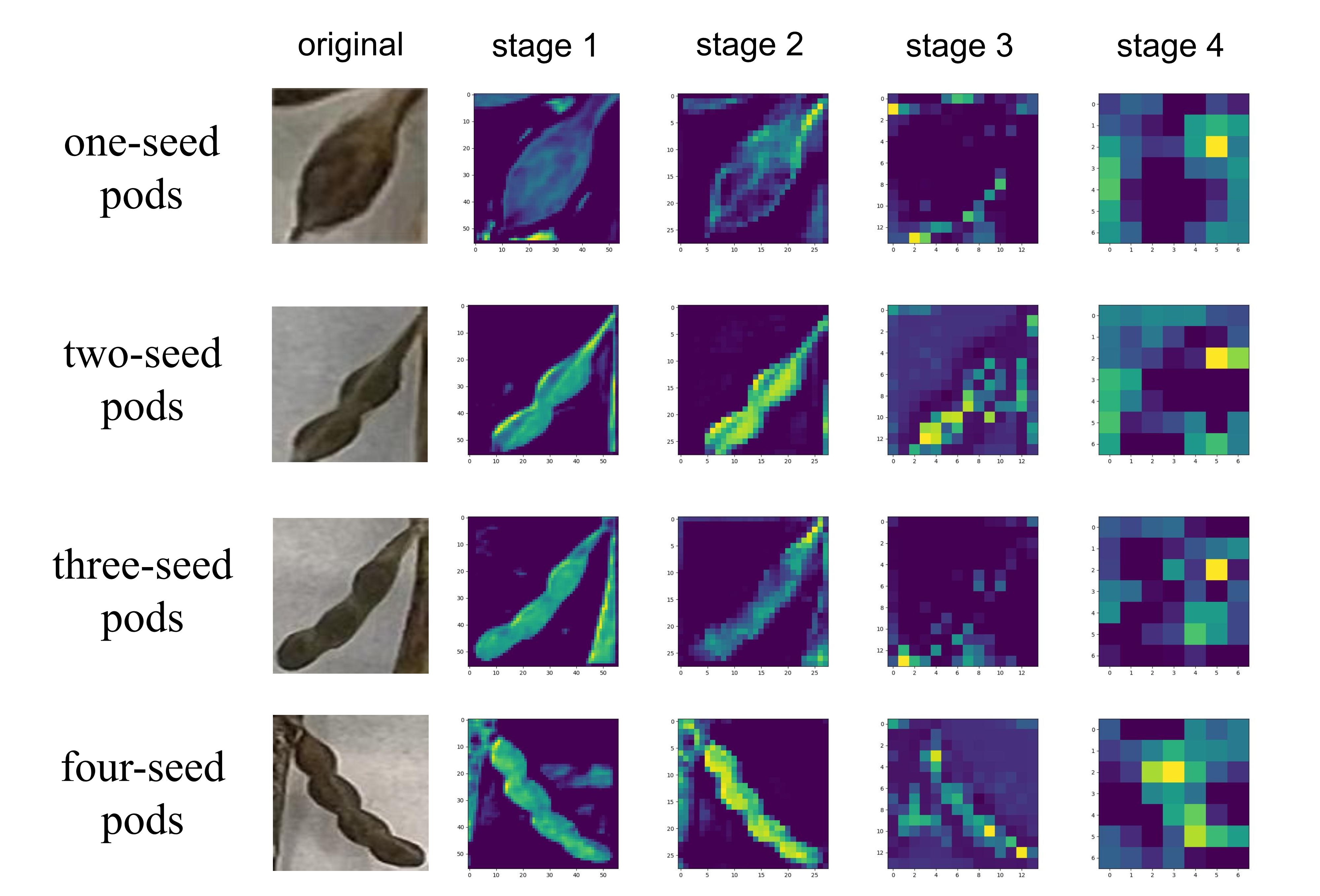

Supplement: Supplementary Figure 4 — The transformation process of different types of pods through feature extraction network. [file Image4.tiff]
