# Supplementary material for: DLML-PC: an automated deep learning and metric learning approach for precise soybean pod classification and counting in intact plants
Source: Front Plant Sci. 2025 Jul 21;16:1583526. doi: 10.3389/fpls.2025.1583526 (PMC12319039; doi:10.3389/fpls.2025.1583526)
Supplement: Supplementary Figure 2 — A note on supplementary counting through metric learning. [file Image2.pdf]

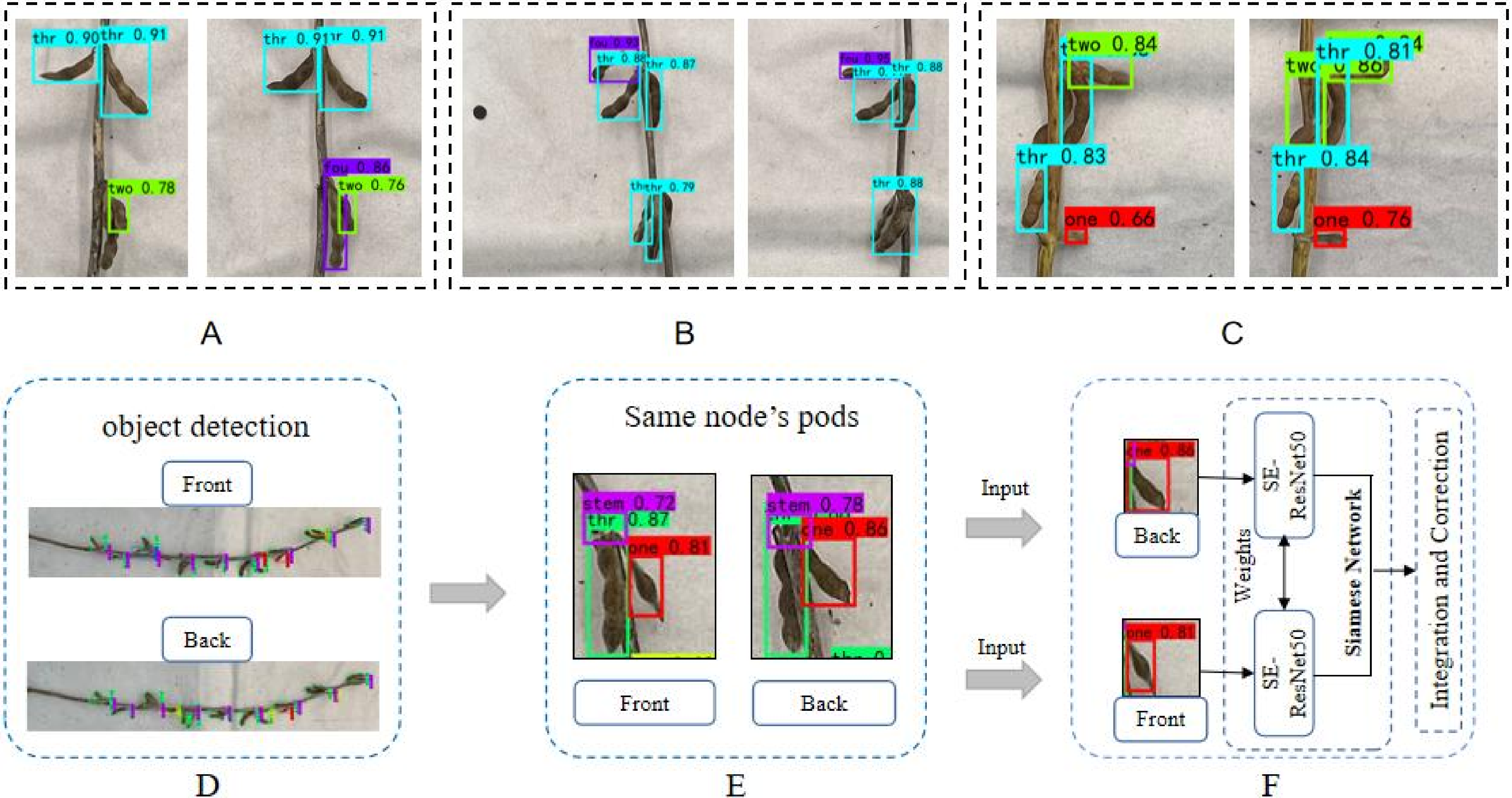

thr 0.90
thr 0.91
two 0.78
thr 0.91
fou 0.86
two 0.76
thr 0.87
thr 0.79
thr 0.88
thr 0.88
two 0.84
thr 0.83
one 0.66
thr 0.81
thr 0.84
one 0.76
A
B
C
object detection
Front
Back
Same node's pods
stem 0.72
thr 0.87
one 0.81
stem 0.78
one 0.86
Front
Back
Input
Input
Back
Front
Weights
SE-ResNet50
SE-ResNet50
Siamese Network
Integration and Correction
D
E
F
