# Supplementary material for: DLML-PC: an automated deep learning and metric learning approach for precise soybean pod classification and counting in intact plants
Source: Front Plant Sci. 2025 Jul 21;16:1583526. doi: 10.3389/fpls.2025.1583526 (PMC12319039; doi:10.3389/fpls.2025.1583526)
Supplement: Supplementary Table 1 — Experimental environment settings. [file Table1.docx]

Table S1 Experimental environment settings

| Hardware | Software |
| --- | --- |
| 12th Gen Intel (R) Core (TM) i9-12900KF CPU | Windows10 |
| NVIDIA 3090 (24G) GPU | Python3.6 |
| 64G RAM | Pytorch1.10 |
