# Supplementary material for: DLML-PC: an automated deep learning and metric learning approach for precise soybean pod classification and counting in intact plants
Source: Front Plant Sci. 2025 Jul 21;16:1583526. doi: 10.3389/fpls.2025.1583526 (PMC12319039; doi:10.3389/fpls.2025.1583526)
Supplement: Supplementary Table 2 — Experimental parameter settings. [file Table2.docx]

Table S2 Experimental parameter settings

| Name | Hyper parameter | Value |
| --- | --- | --- |
| Faster R-CNN (ResNet50) | optimizer | Adam |
|  | Initial learning rate | 1e-4 |
|  | Momentum | 0.9 |
| Faster R-CNN (VGG16) | optimizer | Adam |
|  | Initial learning rate | 1e-4 |
|  | Momentum | 0.9 |
| CenterNet | optimizer | Adam |
|  | Initial learning rate | 5e-4 |
|  | Momentum | 0.9 |
| SSD | optimizer | SGD |
|  | Initial learning rate | 2e-3 |
|  | Momentum | 0.937 |
| YOLOX | optimizer | SGD |
|  | Initial learning rate | 1e-2 |
|  | Momentum | 0.937 |
| YOLO v7 | optimizer | SGD |
|  | Initial learning rate | 1e-2 |
|  | Momentum | 0.937 |
| RetinaNet | optimizer | Adam |
|  | Initial learning rate | 1e-4 |
|  | Momentum | 0.9 |
| YOLO v5 | optimizer | SGD |
|  | Initial learning rate | 1e-2 |
|  | Momentum | 0.937 |
