# Supplementary material for: DLML-PC: an automated deep learning and metric learning approach for precise soybean pod classification and counting in intact plants
Source: Front Plant Sci. 2025 Jul 21;16:1583526. doi: 10.3389/fpls.2025.1583526 (PMC12319039; doi:10.3389/fpls.2025.1583526)
Supplement: Supplementary Table 3 — Effects of different hyperparameters on accuracy. [file Table3.docx]

Table S3 Effects of different hyperparameters on accuracy

| Batchsize | Optimizer | Lr | Accuracy |
| --- | --- | --- | --- |
| 32 | Adam | 0.01 | 88.4 |
|  |  | 0.001 | 93.1 |
|  | SGD | 0.01 | 85.5 |
|  |  | 0.001 | 83.3 |
| 16 | Adam | 0.01 | 92.8 |
|  |  | 0.001 | 92.9 |
|  | SGD | 0.01 | 86.3 |
|  |  | 0.001 | 85.1 |
| 8 | Adam | 0.01 | 87.6 |
|  |  | 0.001 | 86.5 |
|  | SGD | 0.01 | 84.7 |
|  |  | 0.001 | 82.3 |
